# GAD Antibodies as Key Link Between Chronic Intestinal Pseudoobstruction, Autonomic Neuropathy, and Limb Stiffness in a Nondiabetic Patient: A CARE-Compliant Case Report and Review of the Literature

**DOI:** 10.1097/MD.0000000000001265

**Published:** 2015-08-07

**Authors:** Andrea Maier, Vera Mannartz, Hermann Wasmuth, Christian Trautwein, Ulf-Peter Neumann, Joachim Weis, Joachim Grosse, Matthias Fuest, Max-J. Hilz, Joerg B. Schulz, Christina Haubrich

**Affiliations:** From the Department of Neurology (AM, VM, JBS, CH), University Hospital RWTH Aachen,; Department of Medicine (HW), Luisenhospital Aachen; Department of Gastroenterology (CT), University Hospital RWTH Aachen; Department of Visceral Surgery (U-PN), University Hospital RWTH Aachen; Institute of Neuropathology (JW), University Hospital RWTH Aachen; Department of Urology (JG), University Hospital RWTH Aachen; Department of Ophthalmology (MF), University Hospital RWTH Aachen); and Department of Neurology (M-JH), Erlangen University Hospital, Germany.

## Abstract

Chronic intestinal pseudoobstruction (CIP) can be a severe burden and even a life-threatening disorder. Typically, several years of uncertainty are passing before diagnosis. We are reporting the case of a young woman with a decade of severe, progressive gastrointestinal dysmotility. Unusually, she had also developed an autonomic neuropathy, and a stiff limb syndrome.

In addition to achalasia and CIP the young woman also developed neuropathic symptoms: orthostatic intolerance, urinary retention, a Horner syndrome, and lower limb stiffness. Careful interdisciplinary diagnostics excluded underlying infectious, rheumatoid, metabolic or tumorous diseases.

The detection of GAD (glutamic acid decarboxylase) antibodies, however, seemed to link CIP, autonomic neuropathy, and limb stiffness and pointed at an autoimmune origin of our patient's complaints. This was supported by the positive effects of intravenous immunoglobulin. In response to this therapy the body weight had stabilized, orthostatic tolerance had improved, and limb stiffness was reversed.

The case suggested that GAD antibodies should be considered in CIP also in nondiabetic patients. This may support earlier diagnosis and immunotherapy.

## INTRODUCTION

Chronic intestinal pseudoobstruction (CIP) is a rare condition with symptoms and signs suggestive of mechanical obstruction while a true anatomical lesion is absent. In Japan for instance, Iida et al have found CIP to occur with a prevalence of 1:100.000 with a 2:1 female/male ratio.^1^ CIP is a severe burden as the defective anterograde propulsive activity hinders adequate nutrition, causes weight loss, and may threaten a patient's life. This disease entity typically stays unrecognized for long periods of time before the correct diagnosis is established. In the interim, patients often undergo extensive and repeated diagnostic tests and frequently undergo unnecessary surgery.^2^ Little is known about the time course, disease progression, and the spectrum of disorders related to CIP. We are reporting the case of a 38-year-old nondiabetic young woman, which appeared to be unique due to a slow progression of severe gastrointestinal dysmotility, a subsequent failure of cardiovascular, sudomotor, urinary, autonomic functions, and the late onset of limb stiffness.

## CONSENT

Written informed consent was obtained from the patient for publication of this case report and any accompanying images. A copy of the written consent is available for review by the Editor of this journal.

## CASE REPORT

**FIGURE 1:**
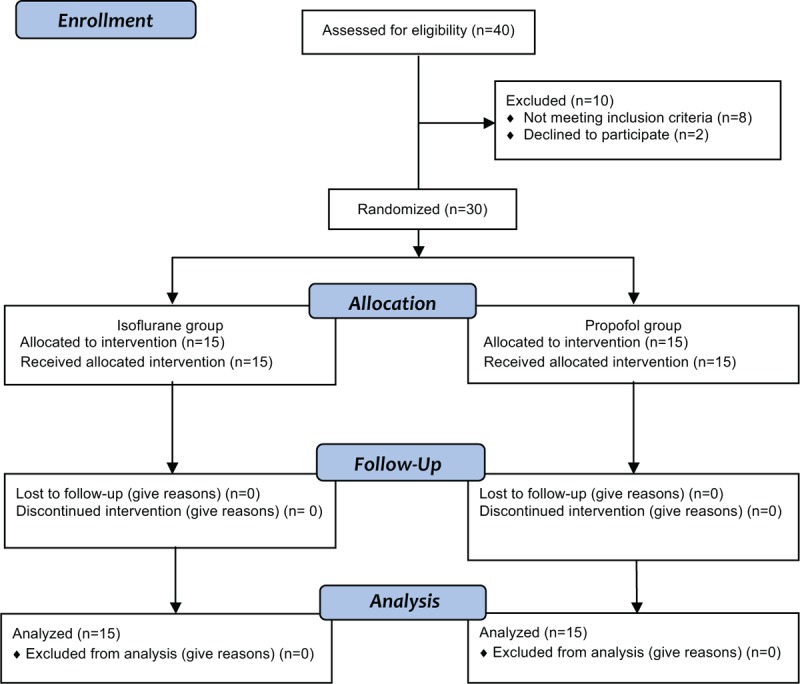
Ophthalmologic, urologic, gastroenterological, tilt table, and MRI imaging. A: Anisocoria right < left before (left image) and 1 hour after 5% cocaine-HCl (right image); B: videourodynamics: detrusor hypocontractility and urinary retention at 15 (left: 600 mL) and 60 minutes (right: 800 mL) after micturition; C: colonic pseudoobstruction 2 (left) and 8 hours (right) after barium meal; D: tilt-table testing with abnormal rise in heart rate (fat line +44 bpm) before (left) and with normal heart rate increase (+16 bpm) after IvIg therapy (right). Systolic and diastolic pressures are shown by thin lines. Vertical lines are marking the period of upright tilt. E: Normal spinal MRI scan, thoracolumbar (left) and cervicothoracal (right); F: Normal pontine plane of cranial MRI scan.

First symptoms began at the age of 28 when achalasia and early satiety were requiring more than 5 meals per day. Constipation with intervals of 3 or more days led to the use of laxatives. After several times of fainting, she had to avoid standing upright for more than 15 minutes. Severe gastroesophageal dysmotility and dysphagia led to a weight loss of 15 kg in 1.5 years. Multiple surgeries followed, for example, gastric fundoplication and esophagectomy in order to enable the gastro-intestinal passage. However, keeping her body weight above 50 kg (height 176 cm) remained difficult. Neither any of her 6 siblings nor her parents had similar symptoms. At the age of 36 years, intestinal dysmotility had advanced to CIP with a complete paresis of the intestinal passage accompanied by severe abdominal pain. Hence, she was instrumented with a percutaneous enteral tube and a stoma. Due to abnormal urinary retention she had to catheterize herself 4 to 6 times daily. Dizziness and palpitations had reduced her orthostatic tolerance to less than 10 minutes. In addition, she had developed dry eyes, dry mouth, dry, irritable skin with recurrent eczema, and difficulties in visual adaptation to darkness. Occasionally she felt paraesthesia and pain in her legs. When she presented in our autonomic clinic at the age of 37 she was emaciated and suffered also from spasms in her right leg. Except for an anisocoria of 1 mm right < left eye and a reduced dilation of the right pupil in the dark (Figure 1A) other cranial nerves were intact. Sensation was normal, including pain, light touch, vibration, and proprioception. Deep tendon reflexes mainly of the right leg were increased. Moving her legs passively was painful and difficult as the leg muscle tone was increased. A paresis or abnormal plantar responses could not be detected.

Testing of the autonomic nervous functions revealed a right-sided Horner syndrome of postganglionic origin upon conjunctival 5% cocaine-hydrochloride stimulation. Schirmer test revealed a bilateral sicca syndrome. Electromyographic cystometry showed a detrusor hypocontractility with urinary retention (Figure 1B). Gastrointestinal radiographic studies revealed an enormous delay in the Barium enema passage (Figure 1C). Galvanic skin responses were delayed in hands and feet bilaterally (Table 1). Head-up tilting (70°) revealed a postural tachycardia with a heart rate increasing by 35 bpm. The time upright was limited to 5:30 minutes due to presyncopal complaints (Figure 1D). Nerve-conduction studies including somatosensory and motor evoked potentials were normal. Repeated neuromuscular stimulation revealed neither a decrement nor an increment so there were no hints for myasthenia or Lambert Eaton myasthenic syndrome. There were, however, spontaneous grouped neuromyotonic discharges of motor units measured in quadriceps and biceps femoris muscles of the right leg, which were supporting the clinical diagnosis of a stiff limb syndrome. A deltoid muscle biopsy showed largely normal histology except for moderately increased muscle fiber caliber variability and moderate glycogen accumulations in muscle fibers. There were no COX (cyclooxygenase)-negative/SDH (succinate-dehydrogenase)-positive muscle fibers. Mitochondrial enzyme activity showed a normal distribution in the NADH (nicotinamid-adenin-dehydrogenase) essay. On electron microscopy, there were no hints for a mitochondriopathy. A full-layer biopsy of the small intestine showed regular mucosa, submucosa, and muscularis. Ganglia of submucosal (Meissner and Schaberdach) and myenteric (Auerbach) plexuses had normal sizes. There was no indication of hyperinnervation, misplaced or dysplastic ganglia cells. Morbus Hirschsprung or neurointestinal dysplasias were excluded. No amyloid depositions could be detected. Skin biopsy showed normal intraepidermal nerve fibre density and only a few axonal swellings. Neither cranial nor spinal MRI showed ischemic, tumorous, or inflammatory lesions (Figure 1E, F). Repeated imaging studies using x-ray, CT scans of the chest and abdomen, as well as colonoscopies did not bring a hint for a tumorous disease.

**TABLE 1:**
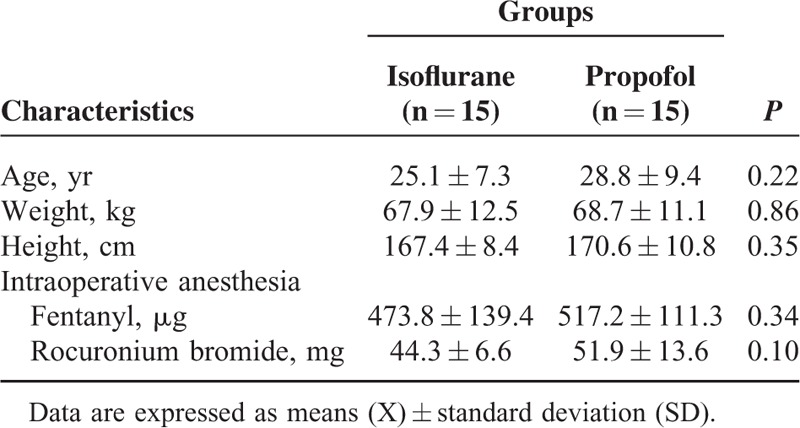
Autonomic Testing Before (Baseline) and During Immunoglobulin Therapy

Routine blood cell counts, blood chemistry, and urine analysis showed no abnormalities. Cerebrospinal fluid examination revealed normal protein and cell count. Serum-glucose, B vitamins (B_1_, B_6_, B_12_), and folic acid were within normal range. The glucose tolerance test was normal as well. Serum tests were negative for infectious diseases (human immunodeficiency virus, hepatitis B and C, lues, borreliosis, herpes zoster, human herpes virus 4, coxsackie virus), endocrine disorders as for instance diabetes mellitus or hypothyreosis, Addison disease, or connective tissue diseases as for instance lupus erythematodes or dermatomyositis. Serologic screening for autoantibodies (Hu, Yo, -Rhi, Ma, Ta, peripherin G, amphiphysin, potassium channel, N-type or P/Q-type calcium channels, GM1, GD1b, GQ1b, muscarinic and nicotinergic acetylcholine receptor antibodies) was negative. Repeatedly, however, were GAD-65 (glutamate decarboxylase isoform 65) antibodies with titers were between 24 and 197 IE/mL (normally <10 IE/mL).

Although the combination of GAD antibody–related limb stiffness, dysautonomia, and CIP appeared to be unique, the literature does provide case examples for each of the above symptoms with autoimmune origin to respond to intravenous immunoglobin (IvIg).^3-5^ We offered this therapy to our patient at intervals of 4 weeks. After 3 cycles of IvIg (0.4 g/kg body weight/day at 5 consecutive days) the orthostatic tolerance was prolonged from standing 5 to 10 minutes and weight had stabilized at 58 kg. Self-catheterization was reduced to 2 times daily. This improvement prevailed for about 3 weeks. Then bladder retention recurred, weight dropped again, gait unsteadiness, and leg muscle stiffness were increasing. Between IvIg cycles 4 and 5, the interval had to be prolonged due to a surgical intervention. When returning to monthly IvIg we had added prednisolone (1 mg/kg/d) in order to maintain the clinical improvement. This could be successively reduced after 3 months. A sustained body weight of 53 kg, a prolonged orthostatic tolerance (32 minutes upright, Figure 1D), and a normalized muscle tone significantly enhanced the patient's life quality and mobility. Gastrointestinal motility and detrusor contractility did improve but did not resolve completely. Hence, the enteral nutrition had to be continued.

## DISCUSSION

CIP may be either secondary to a wide array of recognized pathological conditions or may be idiopathic. Pathophysiological mechanisms of CIP are for instance neurogenic or myogenic.^6^ Myogenic CIP is related to vacuolar degeneration and fibrosis of the muscularis propria and is also known as visceral myopathy.^6^ Neurogenic CIP is classified into 2 forms: inflammatory neuropathies, in which a significant inflammatory/immune response is identified within the myenteric ganglia, and degenerative neuropathies, characterized by evidence of neurodegenerative aspects as altered calcium signaling, mitochondrial dysfunction, and production of free radicals.^6^ CIP can be secondary to diabetes, hypothyroidisms, lupus erythematodes, primary systemic sclerosis, Hirschsprung disease, neoplasia, etc.^2^

Despite extensive diagnostics patients with CIP are often left with uncertainty. In the present case this was true for many years. Finally, a careful interdisciplinary diagnostic workup of the autonomic nervous system including cardiovascular, urinary, secreto-, and pupillomotor functions and limb stiffness implied a neuropathic CIP. The repeated detection of GAD antibodies and the improvement of orthostatic tolerance, sudomotor functions, and limb stiffness, as well as the weight stabilization in response to immunoglobulin therapy suggested an autoimmune pathology in the presented case.

GAD antibodies are considered to be organ specific and are typically associated with diabetes mellitus either type I or the late onset diabetes type II.^7^ GAD antibodies can be found also in nondiabetic patients as in the presented case. The spectrum of GAD antibody related diseases has widened recently and encompasses many more syndromes than diabetes.^8^ Kraichely et al noted the striking frequency of GAD-65 autoantibodies in a cohort of patients with achalasia (11-fold higher than in controls, *P* < 0.0001), which may not be accidental.^9^ This appears related to the fact that γ-amino butyric acid (GABA) is expressed not only in pancreatic β-islet cells but in the GABAergic nerve terminals in the enteric nervous system as well as in the central nervous system.^9^ GAD antibodies are, however, rare findings in patients with predominant enteric dysmotility.^10^ Dhamija et al noticed that gastrointestinal dysmotility in GAD antibody–positive patients is rather occur in coexistence with other autoimmune diseases as hypothyroidism, diabetes mellitus, lupus erythematodes, myasthenia gravis, and others.^11^ The pathophysiological role of GAD-65 antibodies in nondiabetic syndromes is still unclear. In GAD-related gastrointestinal dysmotility hypothetically, antibodies may hinder the conversion of GAD-65 into glutamic acid to GABA, which is expressed in GABAergic nerve terminals in the enteric nervous system.^9,12^ GAD antibodies were also reported in patients with myasthenia gravis, Lambert-Eaton syndrome, autoimmune dysautonomias, autoimmune encephalopathies, and cerebellar ataxia.^8^

Moreover, GAD antibodies can be found in patients with stiff person syndrome or stiff limb syndrome similar to our patient.^9,13^ Unlike the stiff person syndrome that involves truncal musculature as well as signs of brainstem, pyramidal, and sensory dysfunction is the stiff limb syndrome restricted to limb muscles only and is presumed to be due to an autoimmune interneuronitis at the spinal level.^14^ The stiff limb syndrome may be accompanied by diabetes mellitus, thyroid disease, and may be part of paraneoplastic syndromes.^15^ Occasionally, rigidity of the limbs is seen in the setting of breast or small cell lung carcinoma and would typically be associated with amphiphysin antibodies.^15,16^ In our patient, neither were detected amphiphysin antibodies nor have several years of thorough diagnostics brought a hint for a tumor.

Factors that would typically raise the clinical suspicion of autoimmune gastrointestinal dysmotility as antecedent events or a subacute disease onset were absent in the present case. However, IvIg had improved the orthostatic intolerance as well as the sicca syndrome and has led to a weight stabilization. Although gastrointestinal dysmotility and orthostatic intolerance could be improved and the patient regained mobility, the improvement was only partial. The slow progression of autoimmune diseases is supposed to involve both an inflammatory and a degenerative process.^17^ The latter could explain why the improvement of gastrointestinal dysmotility in our patient was only partial.

An earlier detection of GAD antibodies would have enabled a sooner diagnosis and therapy in our patient, which may have possibly postponed the beginning of enteral nutrition. Hence, we suggest that GAD antibodies should be considered also in nondiabetic patients with unclear gastrointestinal dysmotility or CIP.

## CONCLUSION

This report presents the case of a nondiabetic patient with a chronic progressive gastrointestinal pseudoobstruction. Years after the onset of gastrointestinal dysmotility, the patient developed dysautonomia and stiff limb syndrome. The detection of GAD antibodies and the positive response to IVIg had confirmed the autoimmune origin of CIP, which appeared to be part of an autoimmune neuropathy. In order to consider the immunotherapy earlier we suggest to screen for GAD antibodies in patients with CIP.

## References

[1] Iida H Ohkubo H Inamori M Epidemiology and clinical experience of chronic intestinal pseudo-obstruction in Japan: a nationwide epidemiologic survey. *J Epidemiol* 2013; 23:288–294. 23831693 10.2188/jea.JE20120173 PMC3709546

[2] Gabbard SL Lacy BE Chronic intestinal pseudo-obstruction. *Nutr Clin Pract* 2013; 28:307–316. 23612903 10.1177/0884533613485904

[3] Modoni A Mirabella M Madia F Chronic autoimmune autonomic neuropathy responsive to immunosuppressive therapy. *Neurology* 2007; 68:161–162. 17210903 10.1212/01.wnl.0000251194.82212.75

[4] McKeon A Robinson MT McEvoy KM Stiff-man syndrome and variants clinical course, treatments, and outcomes. *Arch Neurol* 2012; 69:230–238. 22332190 10.1001/archneurol.2011.991

[5] Flanagan EP Sayto YA Lennon VA Immunotherapy trial as diagnostic test in evaluating patients with presumed autoimmune gastrointestinal dysmotility. *Neurogastroenterol Motil* 2014; 26 9:1285–1297. 25039328 10.1111/nmo.12391 PMC4149849

[6] de Giorgio R Guerrini S Barbara G New insights into human enteric neuropathy. *Neurogastroenterol Motil* 2004; 16 suppl 1:143–147. 15066021 10.1111/j.1743-3150.2004.00491.x

[7] Lundgren VM Isomaa B Lyssenko V GAD antibody positivity predicts type 2 diabetes in an adult population. *Diabetes* 2010; 59:416–422. 19864397 10.2337/db09-0747 PMC2809967

[8] Saiz A Blanco Y Sabater L Spectrum of neurological syndromes associated with glutamic acid decarboxylase antibodies: diagnostic clues for this association. *Brain* 2008; 131:2553–2563. 18687732 10.1093/brain/awn183

[9] Kraichely RE Farrugia G Pittock SJ Neural autoantibody profile of primary achalasia. *Dig Dis Sci* 2010; 55:307–311. 19499338 10.1007/s10620-009-0838-9 PMC2819289

[10] Törnblom H Lang B Clover L Autoantibodies in patients with gut motility disorders and enteric neuropathy. *Scand J Gastroenterol* 2007; 42:1289–1293. 17918010 10.1080/00365520701396216

[11] Dhamija R Meng Tan K Pittock SJ Serological profiles aiding the diagnosis of autoimmune gastrointestinal dysmotility. *Clin Gastroenterol Hepatol* 2008; 6:988–992. 18599359 10.1016/j.cgh.2008.04.009 PMC2741093

[12] Reis HJ Van den Berghe P Romano-Silva MA Gaba-induced calcium signalling in cultured enteric neurons is reinforced by activation of cholinergic pathways. *Neuroscience* 2006; 139:485–494. 16446040 10.1016/j.neuroscience.2005.12.023

[13] Dalakas MC Fujii M Li M McElroy B The clinical spectrum of GAD-positive patients with stiff-person syndrome. *Neurology* 2000; 55:1531. 11094109 10.1212/wnl.55.10.1531

[14] Brown P Rothwell JC Marsden CD The stiff leg syndrome. *J Neurol Neurosurg Psychiatry* 1997; 62:31–37. 9010397 10.1136/jnnp.62.1.31 PMC486692

[15] Barker RA Revesz T Thom M Review of 23 patients affected by the stiff man syndrome: clinical subdivision into stiff trunk (man) syndrome, stiff limb syndrome, and progressive encephalomyelitis with rigidity. *J Neurol Neurosurg Psychiatry* 1998; 146:633–640. 9810930 10.1136/jnnp.65.5.633 PMC2170335

[16] Dalakas MC Stiff person syndrome: advances in pathogenesis and therapeutic interventions. *Curr Treat Options in Neurol* 2009; 11:102–110. 10.1007/s11940-009-0013-9 19210912

[17] Pimentel R Salgado M Magalhães MJ Chronic intestinal pseudo-obstruction as an expression of inflammatory enteric neuropathy. *Port J Gastroenterol* 2014; 21:254–257.

